# Artificial Intelligence-Enabled DDoS Detection for Blockchain-Based Smart Transport Systems

**DOI:** 10.3390/s22010032

**Published:** 2021-12-22

**Authors:** Tong Liu, Fariza Sabrina, Julian Jang-Jaccard, Wen Xu, Yuanyuan Wei

**Affiliations:** 1 College of Sciences, Massey University, Auckland 0632, New Zealand; 2 School of Engineering and Technology, Central Queensland University, Sydney, NSW 2000, Australia; f.sabrina@cqu.edu.au; 3 Cybersecurity Lab, College of Sciences, Massey University, Auckland 0632, New Zealand; j.jang-jaccard@massey.ac.nz (J.J.-J.); w.xu2@massey.ac.nz (W.X.); y.wei1@massey.ac.nz (Y.W.)

**Keywords:** smart transport system, blockchain, smart contract, artificial intelligence, deep learning, autoencoder, multi-layer perceptron, DDoS

## Abstract

A smart public transport system is expected to be an integral part of our human lives to improve our mobility and reduce the effect of our carbon footprint. The safety and ongoing maintenance of the smart public transport system from cyberattacks are vitally important. To provide more comprehensive protection against potential cyberattacks, we propose a novel approach that combines blockchain technology and a deep learning method that can better protect the smart public transport system. By the creation of signed and verified blockchain blocks and chaining of hashed blocks, the blockchain in our proposal can withstand unauthorized integrity attack that tries to forge sensitive transport maintenance data and transactions associated with it. A hybrid deep learning-based method, which combines autoencoder (AE) and multi-layer perceptron (MLP), in our proposal can effectively detect distributed denial of service (DDoS) attempts that can halt or block the urgent and critical exchange of transport maintenance data across the stakeholders. The experimental results of the hybrid deep learning evaluated on three different datasets (i.e., CICDDoS2019, CIC-IDS2017, and BoT-IoT) show that our deep learning model is effective to detect a wide range of DDoS attacks achieving more than 95% *F*1-*score* across all three datasets in average. The comparison of our approach with other similar methods confirms that our approach covers a more comprehensive range of security properties for the smart public transport system.

## 1. Introduction

The smart public transport system (e.g., buses, taxis, subways, community scooters) is an important part of the development of reliable smart city initiatives as it contributes to improving our mobility and significantly decreasing our carbon footprint. For example, the Internet of Vehicles (IoV) is a distributed network of vehicles that allows vehicles to connect, communicate, and exchange data/information with each other over the internet. The IoV helps in the realization of the smart transportation system as it enables transportation vehicles equipped with sensors, software, and computing nodes to connect and communicate with each other.

The various networks involved in the maintenance of the smart public transport system are vitally important to guarantee reliable operations without disruption from unauthorized attacks [1]. The vulnerabilities from cyberattacks that can alter urgent and critical maintenance data or halt underlying transport infrastructure can bring disastrous consequences.

Technological advances in Artificial Intelligence (AI) (e.g., including both machine learning and deep learning) and blockchain have proven capable to improve safety and efficiency in the smart public transport network. There have been increasing proposals of utilizing blockchain technologies to support different aspects of transport facilities and communication [2,3,4,5,6,7,8,9,10]. However, there is a lack of research on utilizing blockchain technology on the public transport maintenance system to safeguard from integrity attacks that can intercept and make unauthorized changes against sensitive transport maintenance data. However, blockchain alone cannot protect the smart transport system.

The smart transport system is at risk of different categories of cyberattacks. Some of the known types of cyberattacks in the smart transport systems include distributed denial of service (DDoS) attack, ransomware attack, malware attack, Man in the Middle attack, Replay attack, False information attack, wormhole, Phishing attack, Spoofing, Routing attacks, Eavesdropping, etc. [11,12]. In the blockchain context, DDoS has been identified as one of the common attacks often discussed [13]. For example, [14] discussed a DDoS attack launched to block the exchange of smart contracts while [15] described a case where DDoS attacks were attempted in different parts of the blockchain network and blockchain nodes in the smart transport system. To provide ongoing availability of critical infrastructure involved in the blockchain-enabled smart transport system, a more comprehensive approach to detect different categories of cyberattacks is required.

We propose an AI-enabled DDoS Detection model for a blockchain-based public transport system that can withstand various cybersecurity attacks. The blockchain part of our proposed approach enables the protection of the smart transport system from any integrity attacks that attempt to modify sensitive transport maintenance data. The deep learning part of our proposed approach, which combines an autoencoder (AE) as a feature extractor and multi-layer perceptron (MLP) as a classifier, can detect and classify a wide range of DDoS attempts that can potentially block or halt the exchange of urgent and critical maintenance data across the stakeholders of the smart public transport system. The contribution of our proposed approach is following:

- Our proposed approach that combines blockchain and deep learning methods provides comprehensive protection for the smart public transport systems from various cybersecurity attacks;
- The comparison of our proposed approach that effectively combines both blockchain and deep learning methods illustrates that our approach covers a wider range of security properties compared to other similar methods.

We organize the rest of the paper as follows. We examine the related work in Section 2. We provide a smart transport use case in Section 3. We provide the details of the blockchain-based mechanism that protects the integrity of the smart transport system in Section 4. Section 5 provides the details of the hybrid deep learning methods that protect the availability of the smart transport system. In Section 6, the experimental results and comparison with other similar methods are provided. Finally, we provide a conclusion of our work including the future work directions in Section 7.

## 2. Related Work

### 2.1. Blockchain in Transport System

Blockchain technologies can support autonomous vehicles, such as improving security, providing shared storage, optimizing vehicular functionalities. To secure sensitive information of users, vehicles, and reliable data sources, a blockchain-based privacy-preserving scheme for multimedia data sharing in vehicular social networks was proposed [3].

To secure sensitive user information, a blockchain-based privacy-preserving vehicle-to-grid networks payment mechanism was proposed [4]. The smart vehicles may communicate with the stationary edge nodes that could offer blockchain computations and storage. The blockchain can be used to manage self-sovereign identity which people can store and control their own identity data [5].

A blockchain-based secure key management for ensuring communication security was proposed [2]. Balasubramaniam et al. [6] proposed a blockchain-based intelligent transportation system to store road accidents, congestion, delays related data in the blockchain. To reduce the data dimensionality three different techniques, such as Principal Component Analysis (PCA), Linear Discriminant Analysis (LDA), and Non-negative Matrix Factorization (NMF) have been used in this work. This is to ensure that only relevant information goes for the consensus.

Blockchain-based access control architecture for a large-scale IoT-based smart city scenario has been investigated in [16,17]. The authors in [17] looked into the access control in smart transport scenarios where data are shared among users across multiple organizations. To promote seamless access control and maintain consistency of load distribution among distributed roadside units, a blockchain-based vehicular data management framework was proposed [7]. With an effective blockchain tracking system, the vehicles and their status can be easily tracked without the need for a third party. On this count, it improves the payment and dispute resolution of the transportation system [8]. A framework using blockchain technologies to support privacy-oriented, certified and shared transportation information management was proposed [9].

Blockchain technology integrated with AI and ML was explored in promoting safe transport with a surveillance system in a smart city and reducing security issues in future transportation systems [10].

### 2.2. Deep Learning against DDoS Attack

Applying different deep learning models to detect DDoS attacks using binary classification and to categorize DDoS attack types using multi-class classification has been an active area of research in recent years. A bi-directional long short term memory (biLSTM) and Gated Recurrent Units (GRU) were proposed by [18,19] to classify different types of DDoS attacks using CICDDoS2019 achieving over 90% *F*1-*score*. Sanchez et al. [20] and Samom and Taggu [21] proposed a multi-layer perceptron (MLP) model approach for DDoS attack detection and demonstrated that deep learning approaches were more effective compared to shallow machine learning approaches.

Combining two different AI techniques comprised of (shallow) machine learning and deep learning models has been popular in the last few years. Elsayed et al. [22] proposed an intrusion detection system that combines an autoencoder and recurrent neural network (RNN) for DDoS attack detection achieving a 99% *F*1-*score* using binary classification. Javaid et al. [23] proposed a model that combines an autoencoder with a softmax regression-based classifier on an intrusion dataset. The authors in [24] proposed a hybrid approach comprised of autoencoder and isolation forest to achieve 88.98% accuracy. Wei et al. [25] propose a method combines AE and MLP on CICDDoS2019 with *F*1-*score* reaching 98%. Most of these works are limited to evaluating the effectiveness of their proposal only using a single dataset.

In the smart city context, Ferrag et al. [26] proposed an RNN-based deep learning model to detect general intrusion attacks, including some aspects of DDoS attacks, in the smart energy system and evaluates their proposal with three different datasets that include CIC-IDS2017, a power system dataset, and a Bot-IoT dataset achieving as high as 98% accuracy. Zhou et al. [27] proposed a stacked autoencoder model to protect DDoS attacks on the smart grid. Instead of using public datasets, they collected 2 million records of DoS attacks and tested their proposed model achieving 96% of classification accuracy.

## 3. A Smart Transport Use Case

The smart transport system requires many stakeholders to communicate with each other in real-time for the smooth operation of the public transport system. Smooth operations in smart transport systems rely on a series of events. For example, regular maintenance is an important aspect is reliable and timely communication among those who are involved. The main functionalities of the smart transport systems are monitoring regular maintenance of buses, monitoring real-time traffic conditions in specific areas, and locating traffic emergencies (i.e., traffic accidents) in specific areas.

A maintenance scenario within a smart transport system may require the following steps:

- Local smart public transport authority delegates the task to the maintenance service provider;
- Service provider does the regular inspection and generates an event and logs a transaction in the blockchain;
- Maintenance team completes the regular maintenance as the agreement and logs a message once it is completed;
- Maintenance team contacts the supplier if any part is required for the maintenance;
- Once the order is supplied then the maintenance team will make the payment to the supplier;
- Local transport authority can check if the service has been provided as per the Service Level Agreement (SLA);
- Local transport authority will make the payment after the service is provided, and the event will be logged in the blockchain;
- Regular maintenance log will be helpful for other events such as break down, accident, etc.

For example, the maintenance of public transport starts according to the scheduled date and time. During the maintenance, the maintenance team records the detailed activities, including the vehicle number, mechanic’s identification number, starting time, ending time, parts replaced, all aspects of the maintenance or repair activity, and an invoice. The parts replaced will trigger the inventory level updated. The order for the parts is made if the inventory level is too low. The supplier will deliver the parts and issue the invoice to the service provider.

## 4. Blockchain-Based Mechanism

**Figure 1:**
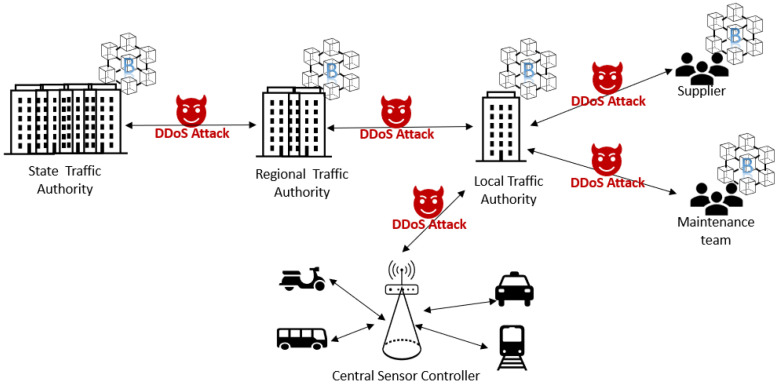
Smart transport architecture.

In this section, we describe our system architecture for a blockchain-based smart public transport maintenance system. The main components of the proposed architecture (as shown in Figure 1) are described below:

Local Transport Authority (LTA): Local transport authority collects the local traffic data from vehicles, roadside units (RSUs), and other entities. It also publishes important data to a blockchain and sends aggregated important information to regional transport authorities. The local transport authority aggregates the local maintenance data and sends it to the regional transport authority.

Maintenance Team (MT): Maintenance team is the external body that has an agreement with the local transport authority for the maintenance of the public transport. They are responsible for regular maintenance. They communicate with supplied if any parts are needed for the maintenance.

Supplier (SP): Supplier has the agreement with the maintenance team and supplies the parts once ordered.

Regional Transport Authority (RTA) and State Transport Authority (STA) are the policymakers and regulatory bodies and can access the maintenance report from blockchain if needed. Regional transport authorities aggregate regional maintenance data and send it to the State Transport Authority.

Blockchain: Each of the main entities (local transport authority, maintenance team, supplier, regional transport authority, and state transport authority) in this system will have a permission blockchain node to record various activities (such as creating a maintenance agreement, job completion, supply order, payment completion, etc.). A smart contract will be deployed within the blockchain which will have the required interface for creating blockchain transactions and reading the necessary information.

As shown in Figure 1, attackers could launch DDoS attacks at different parts of the blockchain network, such as in between the State Traffic Authority and the Regional Traffic Authority, in between the Regional Traffic Authority and the Local Traffic Authority, in between the Local Traffic Authority and the Supplier and Maintenance team, and also in between the local traffic authority and the Central Sensors Controller.

### 4.1. Smart Contract and Algorithms

In this section, we describe the interfaces of the smart contract that is called to create maintenance agreements between the local transport authority and the maintenance team, to log the transaction for completion of the job, log the event of order placement to the supplier, order delivery by the supplier, payment to the supplier, and also payment to the maintenance team. The data are stored as key-value pairs in the Blockchain. We have used JavaScript Object Notation (JSON) data structure for storing the values.

#### 4.1.1. Creating Maintenance Agreement

Algorithm 1 shows how the transaction for agreement (between the local transport authority and the maintenance team) is created in the blockchain. This transaction is logged by the local transport authority. It takes the frequency of maintenance (Freq), Cost, agreement ID (agrID), maintenance team ID (mtID), maintenance contract description (mContractDesc), and service level agreement for the time frame for maintenance (tfSLA) as input parameters and the algorithm returns success or error as the output. Here, the agreement details are written in the blockchain using the putState API. We used putState API in our algorithm as it is used to write on the ledger in the Hyperledger Fabric network. putState API requires a key and a value for its operation. In this case, the agreement key (argKey) and the agreement details (AgrDetails) are the respective key and values.
**Algorithm 1:** Creating Maintenance Agreement **Input**: Freq, Cost, agrID, mtID, mContractDesc, tfSLA **Output**: Success or Error **begin**  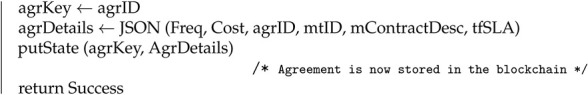
 **end**

#### 4.1.2. Completion of the Job

Algorithm 2 shows how the transaction for the completion of the maintenance job is logged to the blockchain by the maintenance team. Here agreement ID (agrID), maintenance team ID (mtID), maintenance details (mDetails), vehicle ID (vehicleID), a time when the maintenance job came (timeIn), and completion time (timeCompld) is used as input parameters, and the algorithm returns success as output after successfully creating the transaction in the blockchain. We used getState API for reading information such as agreement details (for a given agreement ID), as getState API is used to read states from the ledger in the Hyperledger Fabric network. getState API returns the value for a given key from the state. The job completion details (jcDetails) are written in the blockchain using the putState API and the corresponding key for that is job completion key (jcKey).
 **Algorithm 2:** Completion of the Job **Input**: agrID, mtID, mDetails, vehicleID, Date, timeIn, timeCompleted **Output**: Success or Error **begin**  
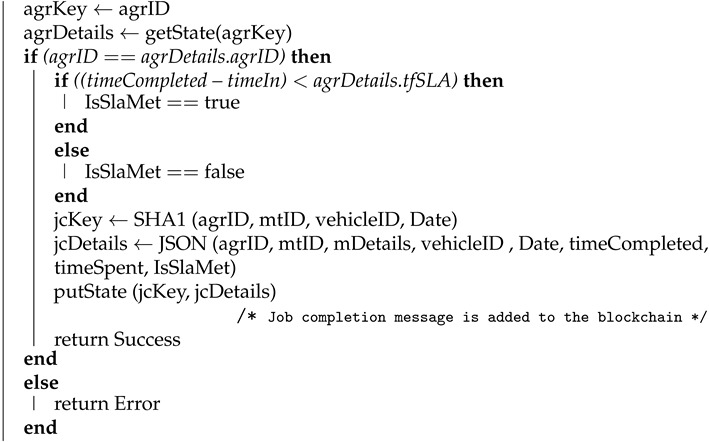
 **end**

#### 4.1.3. Order to the Supplier

Algorithm 3 shows how the transaction for the order placement to the supplier is logged in the blockchain. This transaction is logged by the maintenance team once any parts/tools need to be ordered. Here, order ID (ordID), supplier ID (sID), maintenance team ID (mtID), order description (oDescptn), order date and time (ordDateTime), service level agreement for delivery date/time (deliveryDTSLA), priority level (priorityLevel), and order status (ordStatus) is taken as input parameters. For this function “Ordered” is passed as the parameter for ordStatus. Upon successfully logging in the transaction on blockchain this algorithm returns success as the output. The order details will be stored in the blockchain using the putState API and the corresponding key and value are order key (ordKey) and order details (ordDetails), respectively.
**Algorithm 3:** Order placement to the supplier **Input**: ordID, sID, mtID, oDescptn, ordDateTime, deliveryDTSLA, priorityLevel, ordStatus **Output**: Success or Error **begin**  
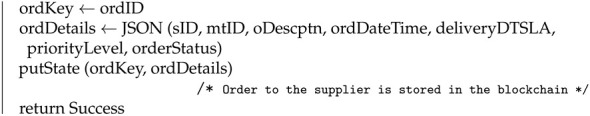
 **end**

#### 4.1.4. Order Delivered

Algorithm 4 shows how the transaction for order delivery is logged by the supplier once the order is delivered. Here, order ID (ordID), delivery date and time (dDateTime), order status (orderStatus), invoice amount (invoiceAmount) are used as input parameters. For this function “delivered” is passed as the parameter for ordStatus. Upon successfully logging in the transaction on blockchain this algorithm returns success as an output. The order delivery details will be stored in the blockchain using the putState API and the corresponding key and value are order key (ordKey) and order details (ordDetails), respectively.
**Algorithm 4:** Order delivered **Input**: ordID, dDateTime, orderStatus, invoiceAmount **Output**: Success or Error **begin**  
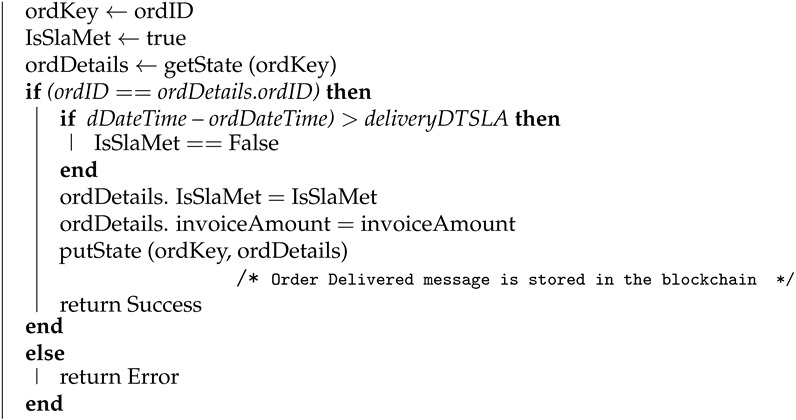
 **end**

#### 4.1.5. Payment to the Supplier

Algorithm 5 shows how the payment completion transaction for the ordered parts is logged in the blockchain. This transaction is logged by the maintenance service once the payment to the supplier is completed. Here, order ID (ordID), payment date and time (payDateTime), and paid amount (paidAmount) is used as input and the algorithm will return success once the transaction is logged on the blockchain. The payment details will be stored in the blockchain using the putState API and the corresponding key and value are order key (ordKey) and order details (ordDetails), respectively.

#### 4.1.6. Payment to the Maintenance Team

Algorithm 6 shows how the payment completion transaction for a maintenance service is logged in the blockchain. This transaction is logged by the local transport authority once the payment for the maintenance service is performed. Here, agreement ID (agrID), payment date and time (payDateTime), and paid amount (paidAmount) is used as input and the algorithm returns success once the transaction is logged on the blockchain. The payment details will be stored in the blockchain using the putState API and the corresponding key and value are agrKey (agreement key) and agrDetails (agreement details), respectively.
**Algorithm 5:** Payment to the supplier **Input**: ordID, payDateTime, paidAmount **Output**: Success or Error **begin**  
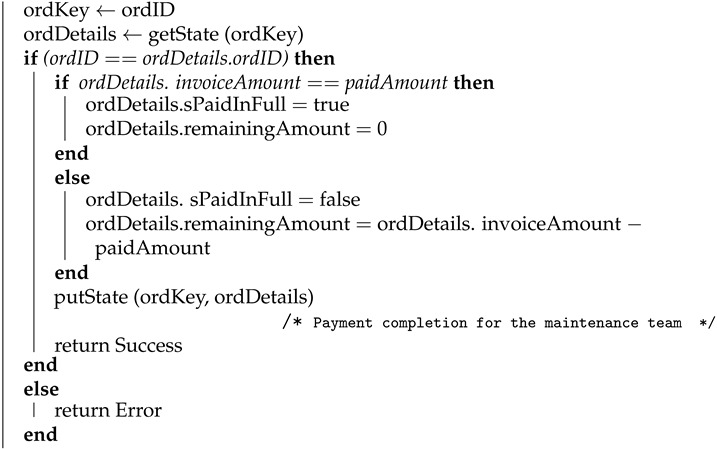
 **end**

**Algorithm 6:** Payment to the Maintenance Team **Input**: ordID, payDateTime, paidAmount **Output**: Success or Error **begin**  
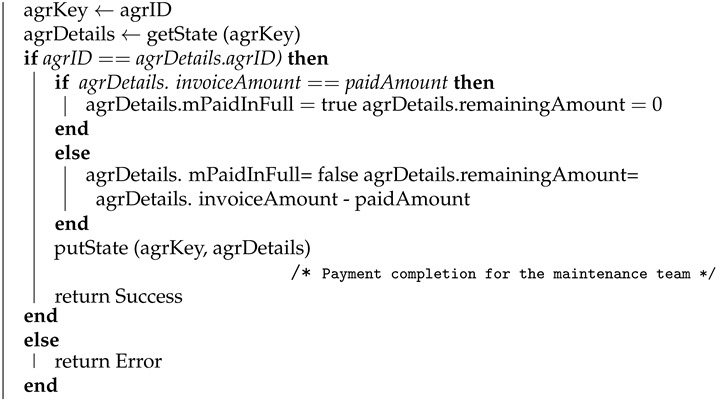
 **end**

### 4.2. Integrity Protection

**Figure 2:**
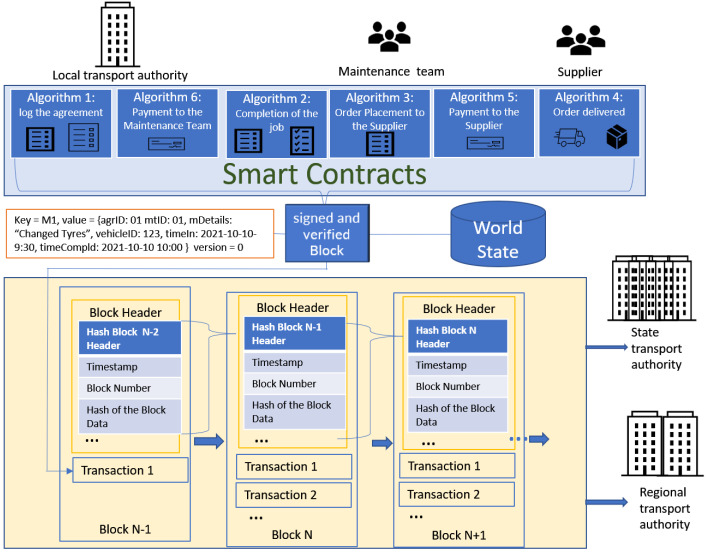
Blockchain and Smart Contract.

The smart contracts are digital versions of agreements among the local transport authority (LTA), the maintenance team (MT), and the supplier (SP). Once the smart contracts are deployed successfully to the blockchain network, they are available to the participants in that network. The regional transport authority (RTA) and state transport authority (STA) can access the network. A smart contract executes on a peer node in the blockchain network. It takes a set of input parameters combines them with its program logic to read and write the block. Once the block is signed and verified, it will be added to the blockchain. The proposed smart contract and blockchain are shown in Figure 2.

The blockchain-based smart public transport maintenance system is composed of a set of five types of nodes connected by a peer-to-peer network.

(1) $$N=\left\{LTA_{i},RTA_{i},NTA_{i},MT_{i},SP_{i}\right\},where\;i=\left\{1,\ldots ,n\right\}$$

The blockchain is maintained by $LTA_{i}$, $MT_{i}$, and $SP_{i}$ nodes while $RTA_{i}$ and $NTA_{i}$ nodes do not participate in the consensus process.

$G_{1}$, $G_{2}$, $G_{T}$ are three groups of prime order *q* where |G${}_{1}|$ = |G${}_{2}|$ = $G_{T}$ = *q*. Given a security parameter *k*, the private key generator (PKG) first chooses Groups $G_{1}$ and $G_{2}$ of prime order $q>2^{k}$. PKG then chooses a generator *P* of $G_{1}$ and a randomly master key $s\in Z_{q}$ and computes the public key $P{}_{p}{}_{u}{}_{b}$ = sP. The cryptographic hash functions of domain and range are

(2) $$H_{1}:H_{2}:{\left\{0,1\right\}}^{*}\to G_{1}and\;H_{3}:{\left\{0,1\right\}}^{*}\to Z_{q}$$

The PKG computes the private key for mtID as $sP_{i}$ for i∈{0,1}, where [26]

(3) $$P_{i}=H_{1}\left(mtID,i\right)\in G_{1}$$

(4) $$P_{w}=H_{2}\left(w\right)\in G_{1}$$

(5) $$c_{1}=H_{3}\left(m_{1},mtID,w\right)\in Z_{q}$$

(6) $$r_{1}\in Z_{q}$$

(7) $$S_{1}=r_{1}P_{w}+sP_{0}+c_{1}sP_{1}$$

(8) $$T_{1}=r_{1}P$$

The signature on M1 is $SigBlock(w,S_{1},T_{1})$. *w* is new string never used before.

As shown in Figure 2, the Key M1 and the data value of agrID: 01, mtID: 01, mDetails: “Changed Tyres”, vehicleID: 123, timeIn: 2021-10-10 9:30, timeCompld: 2021-10-10 10:00 are checked to ensure that the update has complete, the current value of the world state matches the read set of the data when it was signed by $MT_{i}$ and the peer nodes. Then, the data are marked as valid once both these procedures have been completed successfully. The block is added to the blockchain as a transaction.

Each block’s header includes a hash of the prior block’s header, a hash of the current block’s transactions, timestamp, and block number, etc. Since each block contains a hash of the prior block’s header, the blocks on the blockchain are linked together cryptographically. This hashing and linking mechanism make the blockchain data secure. The blockchain is distributed throughout a network. Different nodes store a copy of the blockchain. If a node hosting the blockchain is tampered with, it would not match with all the other nodes, and is almost impossible to convince all the other nodes that the hacked node has the ‘correct’ blockchain.

In Figure 2, Block N-1 has block data which contain the data of completion of the maintenance job stored as transaction 1. The block data were hashed and stored in the header of Block N-1. The hash value of block N-1 header was stored in Block N.

## 5. Deep Learning-Based Mechanism

In this section, we describe the details of a hybrid deep learning model we use and the details of data and data pre-processing methodologies we adapted.

### 5.1. A Hybrid Model

**Figure 3:**
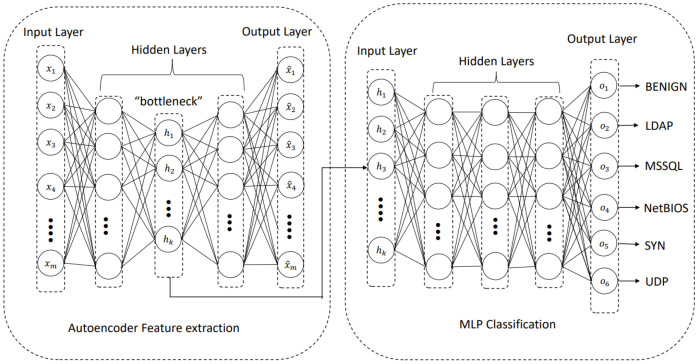
The overview of the hybrid model.

In this study, we use our hybrid approach that combines autoencoder (AE) and multi-layer perceptron (MLP) we developed earlier [25]. We extend our earlier work to include more diverse DDoS attack types that may be used as an attack tool on Smart Transport Systems. In our hybrid approach, we use unsupervised training for the AE model without labels to automate the feature extraction process without human intervention. In contrast, we use supervised learning for the MLP model with labels to evaluate the classification accuracy. We first use autoencoder (AE) as a feature extraction tool [28] to find the most relevant features from the original dataset and then use multi-layer perceptron (MLP) as a classifier that categorizes the attacks into different categories (i.e., classes). The overview of the hybrid approach we use is depicted in Figure 3. A summary of the main components of our model follows.

#### 5.1.1. Feature Extraction

The AE model we use is a feed-forward neural network based on unsupervised learning—that is, the input data does not need to be labeled according to the ground truth.

In an AE model, it tries to reconstruct an output that resembles an input as much as possible. To achieve this goal, AE tries to find and extract the most relevant features from the network traffic samples during training while ignoring the features not directly related to detecting DDoS attacks.

Each time the model is trained, the number of features is reduced (to increase the efficiency of the model) that represents more relevant features. Once the training is complete, only the most relevant features critical for attack detection (and classification) remain in the bottleneck layer (i.e., latent space). Whether the model has found the most relevant features are typically evaluated at the decoding process where the output is reconstructed and its resemblance to the input is evaluated. The model is trained if the difference between the reconstructed output and its corresponding input by computing a reconstruction loss. These can be summarized as follows.

During the encoding phase of an AE, the input *x* is represented as a vector $\left(x\in R^{m}\right)$. This vector is then plotted to the latent space (h). This is shown in Equation (9).

(9) $$h=f_{1}\left(wx+b\right)$$

where $f_{1}$ indicates an activation function for the encoder which outputs the results of computing the weight *w* of each input sample with an additional bias *b*.

During the decoding phase of an AE, the latent space representation (h) is mapped back to reconstruct $\hat{x}$. This is shown in Equation (10):

(10) $$\hat{x}=f_{2}\left(w^{\prime }h+b^{\prime }\right)$$

where $f_{2}$ denotes the decoder’s activation which outputs the results of computing the $w^{\prime }$ of each feature in the bottleneck layer with an additional bias $b^{\prime }$. The $w^{\prime }$ and $b^{\prime }$ are not unrelated to the *w* and *b* used by the encoder.

The reconstruction loss (L) that minimizes reconstruction error on *x* is computed. This is shown in Equation (11).

(11) $$L\left(x,\hat{x}\right)=\frac{1}{n}\sum_{i=1}^{n}{\left(x{}_{i}-\hat{x{}_{i}}\right)}^{2}$$

which *n* indicates the training samples.

#### 5.1.2. Classification

Our MLP contains an input layer, multiple hidden layers, and an output layer. The hidden layer that is trained and has the lowest number of neurons from the AE model is used as an input, represented as the input vector $h_{z}$, to the MLP. The MLP further trains the input vector by producing the vector $y_{z}$ (i.e., latent space representation). The final latent space representation is fed to the output layer to predict different DDoS attack classes. In our model, we use the softmax function for class categorization, as shown in Equation (12).

(12) $$\hat{y}=softmax\left(y_{z}w{}_{y}+b{}_{y}\right)$$

where *w*${}_{y}$ denotes weights while *b*${}_{y}$ denotes bias at the output layer.

#### 5.1.3. Training

The training strategy of the hybrid model is described in Algorithm 7. In our model, the AE is trained in an unsupervised way (i.e., without labeling) to find the most important features while the features that are less important to predict DDoS attacks are discarded (i.e., not represented in the following hidden layer). We divide the input data into several mini-batch to update the weights/bias using Stochastic Gradient Descent (SGD) according to the loss function of Mean Squared Error (MSE) calculation. This is seen in the first for-loop of the Algorithm.

Our 5-layer AE model is designed to have 1 input layer, 2 hidden layers, a bottleneck layer, and 1 output layer. The 2 hidden layers have 32 neurons each while the bottleneck layer has 10 neurons. The feature representations of the input are extracted from the bottleneck layer (i.e., latent space).
**Algorithm 7:** Training of the hybrid model **Input**: Training dataset $X=\{x_{1},x_{2},x_{3},\ldots ,x_{n}\}$ Training Label $Y=\{y_{1},y_{2},\cdots ,y_{n}\}$ Encoder $E_{\phi }$; Decoder $D_{\theta }$; **begin**  
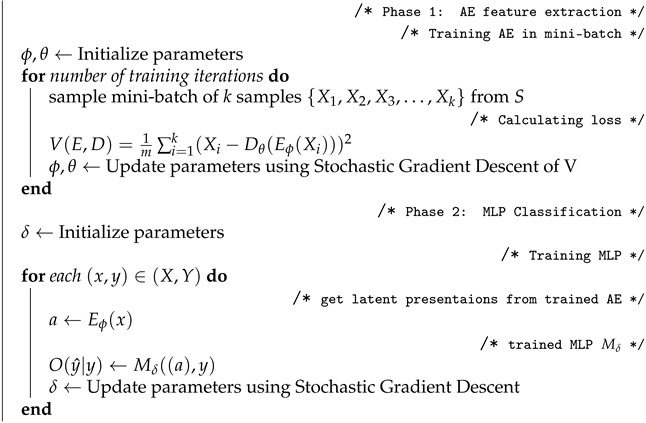
 **end**

The 10 feature representations in the latent space from the AE model are used as the input to the MLP model. This time MLP is trained using the supervised learning method (i.e., using the label) represented in the training dataset. Every input feature is used for the training for updating the weights/bias.

We use a 3-layer MLP model designed to contain: 1 input layer with 10 neurons (i.e., each representing a feature obtained from the latent space of the AE), 1 hidden layer with the size of 32 neurons, and 1 output layer with the number of neurons representing a benign class and other DDoS attack classes. All hidden layers in the hybrid model uses “ReLU” as an activation function while the final output layer of the MLP uses “softmax” as an activation function.

The detailed structures used in the hybrid model are list in the Table 1:

#### 5.1.4. Testing

The testing strategy of the hybrid model is described in Algorithm 8. First, the test dataset is inputted. The most relevant features are extracted and projected in the latent space with the trained AE. Note that the trained AE has the knowledge of which features are most used in a certain DoS attack class. The extracted data, with their label, are then fed into the trained MLP model, which works as a supervised classifier, to identify the specific attack class.
**Algorithm 8:** Testing of the Hybrid model
 **Input**: Testing dataset $X^{\prime }=\left\{x_{1}^{\prime },x_{2}^{\prime },x_{3}^{\prime },\cdots ,x_{n}^{\prime }\right\}$ Testing Label $Y^{\prime }=\left\{y_{1}^{\prime },y_{2}^{\prime },\cdots ,y_{n}^{\prime }\right\}$ **Output**: $O(\hat{y^{\prime }}|y^{\prime })$ **begin**  
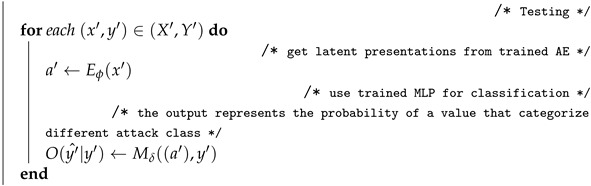
 **end**


### 5.2. Data and Pre-Processing

We discuss the details of the dataset and the pre-processing strategies we used.

#### 5.2.1. Datasets

Considering there are no DDoS datasets yet available specifically exclusive to Smart Transport Systems, we utilize the three largest and most up-to-date DDoS datasets publicly available online. We believe that a comprehensive range of DDoS attack classes, both from reflection-based and exploitation-based DDoS attacks, contained in these datasets are relevant to the Smart Transport Systems context and aligns well with the types of DDoS attack mentioned in existing studies in blockchain [11,12]. We use CICDDoS2019 [29] that contains the largest number of DDoS attack classes that are up to date. We also use CIC-IDS2017 [30] dataset which contains a wide range of intrusion attacks. We only extracted their DDoS attack that exploits HTTP protocol. BoT-IoT dataset [30] contains three different types of attacks but again we only extracted DoS/DDoS attack-related data samples.

These datasets have two different types of sub-datasets, one set is used in the training phase while this other set is used in the testing phase of a deep learning model. The total number of training and testing set categorized whether benign or malicious DDoS payload is shown in Table 2.

The different dataset contains different types of DoS/DDoS attacks. CICDDoS2019 dataset contains different DDoS attack types that exploit a wide range of network or application protocols. We categorize 5 different DDoS attack types that exploit LDAP, NetBIOS, MSSQL, SYN, and UDP flooding.

CIC-IDS2017 dataset contains 4 DoS/DDoS-related attacks, namely ‘DDoS’, ‘DoS GoldenEye’, ‘DoS Hulk’, and ‘DoS slowloris’. These attacks are all HTTP flood attacks obtained either through a live network setup, such as ‘DoS slowloris’ and ‘DoS GoldenEye’, while other attack samples were obtained through network simulation tools such as ‘DoS Hulk’. This dataset distinguishes DoS from DDoS attacks. DDoS attacks are obtained through launching an attack from several attack machines to a target machine while DoS attacks were sent (or simulated) from a single machine to a target machine.

The BoT-IoT dataset only contains 2 different types of attack (DoS and DDoS) that exploits either TCP or UDP protocols.

The summary of different DoS/DDoS attacks and the description of each attack that appears in these three datasets are illustrated in Table 3.

#### 5.2.2. Pre-Processing

We first clean our datasets by removing the features that have been considered irrelevant to classification tasks as suggested by [31]. We further clean the datasets by removing feature values that have no meaning for training, for example, blank or garbage values.

The next step in data pre-processing is to code categorical values to floats/numeric values as deep learning methods cannot work with character values. We either use Label Encoding to encode labels (e.g., benign or indicating specific attack type), or One-Hot-Encoding to encode any categorical/object values to floating/numeric values.

These cleaned and converted input data are now normalized to eliminate the impacts of high variance of feature values. We use min–max-based data normalization (i.e., put values all in the [0,1] range) for the feature scaling.

We also apply either/both Random Undersampling or/and Synthetic Minority Oversampling Technique (SMOTE) [32] to balance training sets that have the imbalanced distribution of samples. For example, the number of training samples in “DoS GoldenEye” (only 7742 records) and “DoS slowloris” (only 4347 records) were significantly under-sampled compared to other attack classes in the CIC-IDS2017 dataset.

**Figure 4:**
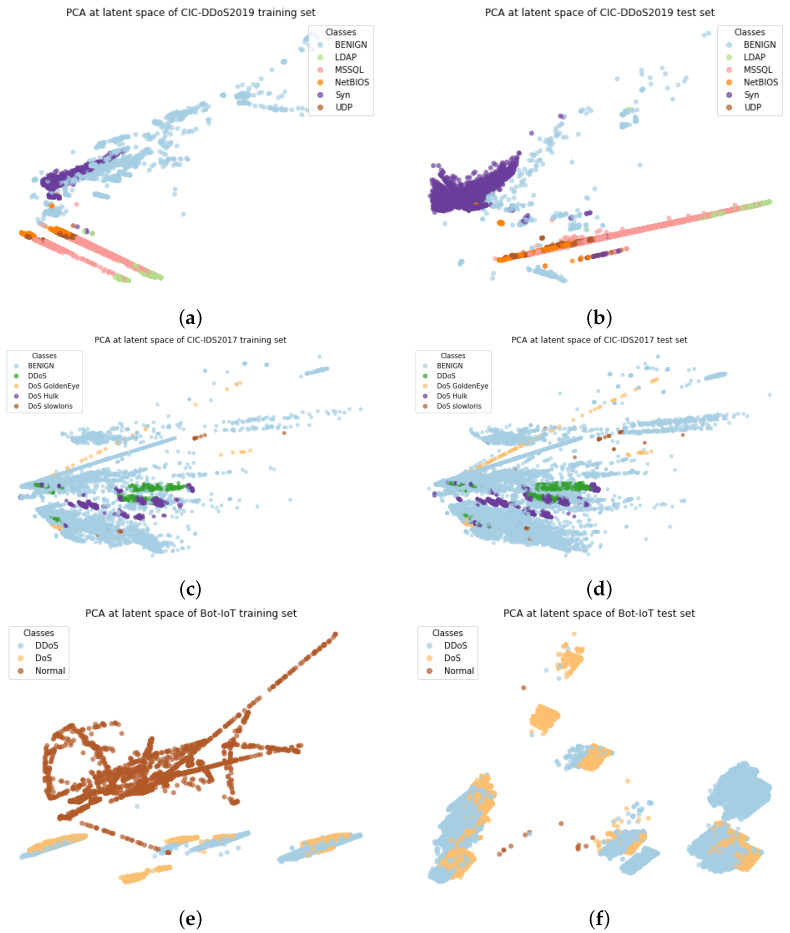
PCA visualization of data distribution of datasets. (**a**) CICDDoS2019 Training set; (**b**) CICDDoS2019 Test set; (**c**) CIC-IDS2017 Training set; (**d**) CIC-IDS2017 Test set; (**e**) Bot-IOT Training set; (**f**) Bot-IOT Test set.

Figure 4 illustrates the PCA-based visualization outcomes of the latent presentation for both the training set (after sampling) and the test set.

In the CICDDoS2019 dataset (Figure 4a,b), the data distribution of “BENIGN” samples both in training and testing sets are significantly more extensive while the data distribution of 5 different DDoS attack types is condensed around smaller clusters.

The data distribution of the CIC-IDS2017 dataset is comparatively more realistic than the CICDDoS2019 dataset as the “BENIGN” samples are dominant in the dataset. This is consistent with the real network where most of the traffic is normal. Meanwhile, the data distribution across different DDoS attack types in CIC-IDS2017 is relatively unbalanced compared to the CICDDoS2019. Due to the above reasons, we decide not to apply the sampling technique on CIC-IDS2017 and we split the dataset into a 3:1 ratio with stratification as no suggestion was proposed by the dataset creators. Therefore, the training set (Figure 4c) and test set (Figure 4d) share the same distribution among the classes.

The training set of the BoT-IoT dataset (Figure 4e) contains significantly large benign samples (after oversampling) with a few clusters of the mix of DoS and DDoS attack samples are formed away from benign samples. Very different from the training set, the test set of the BoT-IoT dataset (Figure 4f) has significantly larger attack samples of DoS/DDoS.

## 6. Evaluation and Analysis

In this section, we describe the details of our setup environment that we used to run our evaluation, the performance metrics we used to measure the effectiveness of the hybrid model, and the experimental results with analysis.

### 6.1. Evaluation Setup

Our hybrid model was built with Tensorflow 2.6 [33] and Keras API [34], and our experiments were carried out on Kaggle.com platform using the following hyper-parameters shown in Table 4.

### 6.2. Evaluation Metrics

We use four performance metrics, recall, precision, *F*1-*score*, and ROC curve, respectively. True Positive (*TP*) indicates the classification that actually positive and predicted positive, True Negative (*TN*) indicates the classification that is actually negative and predicted to negative, False Positive (*FP*) indicates the classification that is actually negative but predicted to positive, and False Negative (*FN*) indicates the classification that is actually positive but predicted to negative.

True Positive Rate (*TPR*), also known as Recall, is used to measure the ratio of truly correct positive prediction compared to the total number of supposed to be positive predictions, as shown in Equation (13).

(13) $$TPR\left(Recall\right)=\frac{TP}{TP+FN}$$

False Positive Rate (FPR) is used to measure the ratio of truly correct negative prediction compared to the total number of supposed to be negative predictions, as shown in Equation (14).

(14) $$FPR=\frac{FP}{FP+TN}$$

*Precision* is used to estimate the ratio of truly correct positive prediction compared to the total number of positive prediction results which is shown in Equation (15).

(15) $$Precision=\frac{TP}{TP+FP}$$

*F*1-*score* is used as the harmonic mean between the precision and the recall which we use as a way to understand the accuracy of the classification accuracy. This is shown using Equation (16).

(16) $$F1\text{-}score=2\times \left(\frac{Precision\times Recall}{Precision+Recall}\right)$$

The area under the receiver operating characteristics (ROC) Curve (AUC-ROC) is used to plot the balance between TPR and FPR using Equation (17).

(17) $$AUC_{ROC}=\int_{0}^{1}\frac{TP}{TP+FN}d\frac{FP}{TN+FP}$$

### 6.3. Evaluation Results

We illustrate different aspects of our evaluation results and analysis.

#### 6.3.1. Results Based on Performance Metrics

Due to the data size, we used 5% of random samples from both CICDDoS2019 and BoT-IoT datasets while all data from CIC-IDS2017. We used 80% of the sampled data to train the model while the rest 20% was used as a validation set further tuning the hyper-parameters. The classification performance based on the performance metrics we defined earlier on the three datasets is shown in Table 5. The performances of all three datasets produce very stable results with high *F*1-*scores*. However, the best performance dataset was from the CICDDoS2019 dataset were two types of DDoS attacks, related to NetBIOS and SYN flood, achieving 100% *F*1-*score* while other classes achieved more than 95%. The result of the BoT-IoT dataset was compatible with the CICDDoS2019 dataset achieving the range from 94% to 100% accuracy. The results from the CIC-IDS2017 dataset had a slight fluctuation with the unbalanced classes, such as DoS slowloris, despite upsampling technique applied, had the lowest *F*1-*score* at 84% while all other classes achieved more than 90%.

These results show the hybrid model we used was well trained without underfitting or overfitting towards any particular DDoS attack type. The result confirms that this type of deep learning model can be an effective mechanism to protect the smart transport system (and other similar systems) against different types of DDoS attacks.

#### 6.3.2. Results Based on Confusion Metrics

**Figure 5:**
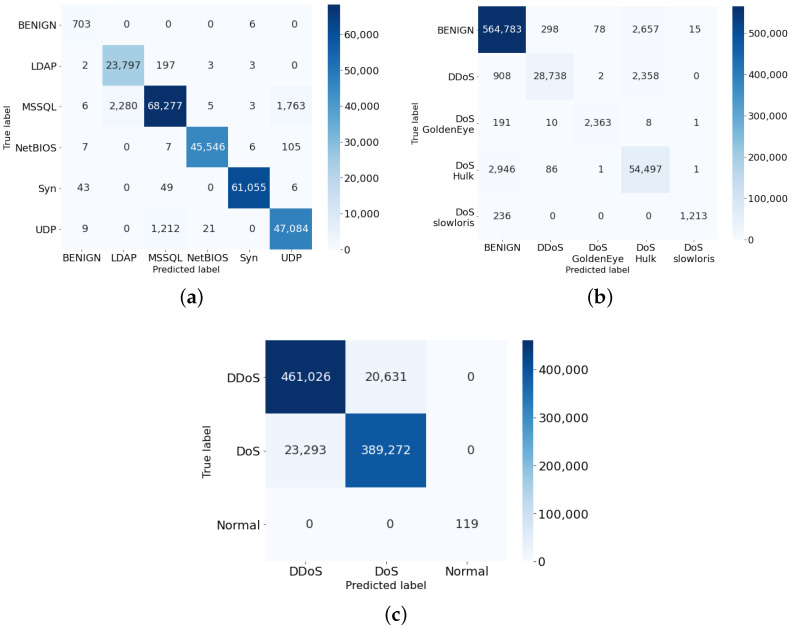
Confusion matrices results on the three test sets. (**a**) CICDDoS2019; (**b**) CIC-IDS2017; (**c**) Bot-IOT.

Figure 5 illustrates the confusion matrix-based classification for different attack types with the exact number of predictions are shown. Both TP and TN rates correctly classify benign and different attack types are very high while FP and FN rates stay very low across all three datasets (i.e., highlighted by different color squares). Though we do not see any color square around True Negative (TN) cases in the benign samples tested on the CICDDoS2019 and the BoT-IoT datasets, it is because they contain a significantly smaller number of benign samples. We also witness the same result in the ‘DoS goldenEye’ attack type when its test samples are significantly smaller in size compared to other classes. We see False Positive (FP) and False Negative (FN) slightly highlighted in the CICDDoS2019 and the BoT-IoT datasets, however, if we take into account the proportion of the FP + FN, they are almost negligent in the scale of a few thousand (e.g., MSSQL/LDAP, MSSQL/UDP) or 10 thousand (e.g., DoS/DDoS, DDoS/DoS) among millions of samples tested, less than 1%.

#### 6.3.3. Results Based on ROC Curves

**Figure 6:**
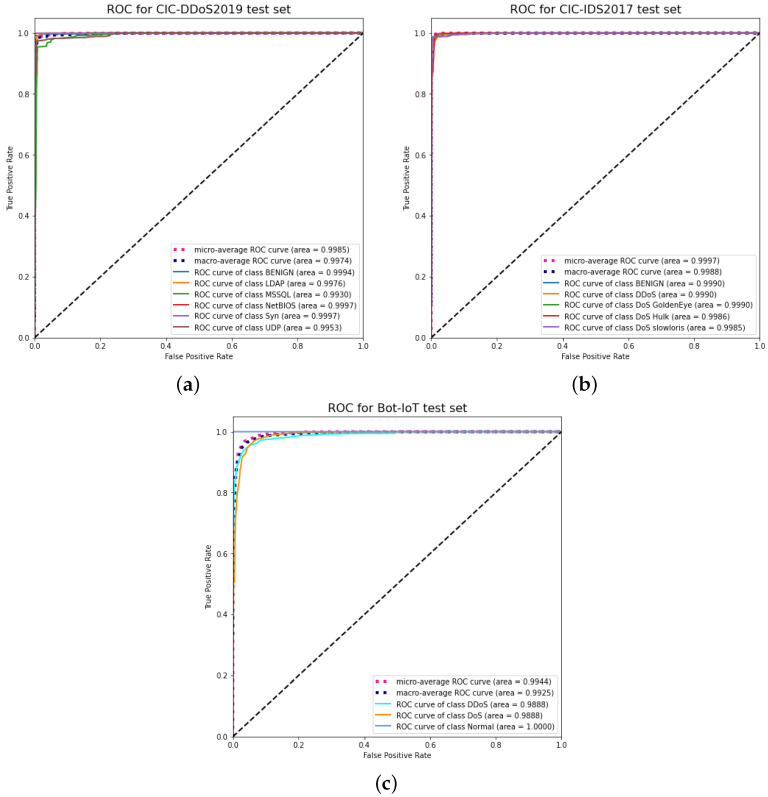
ROC curve results on three test sets. (**a**) CICDDoS2019; (**b**) CIC-IDS2017; (**c**) Bot-IOT.

We also produced ROC curves on our datasets, shown in Figure 6, to analyze the trade-off between True Positive Rate (TPR) and False Positive Rate (FPR) for all classes. Both the micro and macro average of all the attack classification across all datasets are above 0.99. This indicates that the hybrid model has a good detection and classification of malicious payloads containing DDoS attacks.

### 6.4. Comparison between Our System and Other Related Frameworks

Table 6 compares our system with other previously proposed work in the smart transport area. First, we considered if the proposed work is blockchain-based and used smart contracts or not. We also considered data security, integrity, and trust as the metrics for the comparison. Our blockchain-based smart transport system uses smart contracts which are the digital contracts used among the participating entities (such as local transport authority, maintenance team, supplier, etc.). In this scenario, there is no need for any other third party’s involvement and all records of transactions are encrypted and shared only with the authorized participants, this ensures the security and trustworthiness of the information/data. In the blockchain, data are hashed before being added to a block in the chain and that ensures data integrity. Other existing works that we mentioned in Table 6 are also blockchain-based systems and incorporate security, trust, and integrity in their framework. However, one major advantage of our system is that it has incorporated an AI-based Network Intrusion Detection/Prevention system (IDS/IPS) which is highly efficient in detecting different categories of Distributed Denial of Service (DDoS) attacks to ensure the network is not disrupted by the attackers and always available for the intended users. Hence, our system achieves the availability of data/information when it is needed.

## 7. Conclusions

We proposed a comprehensive approach to protecting the smart transport system using the combination of blockchain and deep learning approaches. The blockchain approach in our proposed approach enables sensitive data such as maintenance data to be protected from unauthorized modification (i.e., integrity attack). Using a use case, we provide the details of system architecture, algorithms involved in the smart contracts, and mechanisms to protect the block containing various maintenance data and transactions associated with it. To provide protection from availability attacks (e.g., through DDoS), we add a hybrid deep learning method in our proposed approach to detect various DDoS attacks. In our hybrid deep learning method that combines autoencoder (AE) with multi-layer perceptron (MLP) provides automated feature extraction and classification in a timely manner. The experimental results were obtained through an extensive evaluation of three different datasets namely CICDDoS2019, CIC-IDS2017, and BOT-IoT, that cover a wide range of different DDoS attack types that are most up-to-date. Our extensive experiments show that our proposed approach is effective to detect and classify different types of DDoS attack types without overfitting/underfitting towards any particular DDoS attack type, achieving very high *F*1-*score* rates that exceed 95% on an average of all three datasets. The comparison between our proposed approach and other similar blockchain-based approaches [3,4,6,7] shows that we provide a comprehensive range of security properties (e.g., security, trust, integrity, and availability). This confirms that our approach that combines both blockchain and deep learning methods can be an effective mechanism to protect the smart transport system from various cyberattacks. For future work, we plan to investigate other deep learning approaches for protecting the smart transport system from other types of intrusions, such as network intrusion [36], malware [37,38], and ransomware attacks [39]. We also plan to study a blockchain-based public transport system with the integration of edge computing. 

## Figures and Tables

**Table 1:** Detailed structure used in proposed hybrid model.

Model	Structure Details	Value
AE	Hidden Layer 1 neurons	32
	Hidden Layer 1 Activation Function	ReLU
	Hidden Layer 2 neurons	10
	Hidden Layer 2 Activation Function	ReLU
	Hidden Layer 3 neurons	32
	Hidden Layer 3 Activation Function	ReLU
	Loss Function	MSE
MLP	Hidden Layer 1 neurons	32
	Hidden Layer 1 Activation Function	ReLU
	Output Layer Activation Function	Softmax
	Loss Function	Cross-Entropy

**Table 2:** Record numbers.

Dataset	Phase	Benign	Malicious
CICDDoS2019	Training	56,863	50,006,249
	Test	56,965	20,307,560
CIC-IDS2017	Training	1,703,489	284,806
	Test	567,831	94,934
BoT-IoT	Training	358	2,682,662
	Test	119	894,222

**Table 3:** Summary of DoS/DDoS attacks in our datasets and their descriptions.

Attack Type	Attack Description	Datasets
LDAP Attack	It refers to DDoS attack associated with exploiting Lightweight Directory Access Protocol (LDAP) protocol. The attacker sends a massive number of LDAP requests to the vulnerable LDAP servers by pretending to be a legitimate LDAP client using spoofed IP addresses. The LDAP server becomes too busy to create responses for attackers and becomes unable to respond to real LDAP clients.	CICDDoS2019
NetBIOS Attack	In this attack, attackers exploit the vulnerability associated with the Network Basic Input/Output System (NetBIOS) which is the main protocol used in file-sharing applications. The attackers send a large number of spoofed messages to interfere with all NetBIOS network traffic.	CICDDoS2019
MSSQL Attack	In this attack, attackers exploit the vulnerabilities in Microsoft Structured Query Language (MSSQL) which is used to obtain records from the underlying database. The attacker executes the scripted MSSQL requests to the MSSQL Server to block other legitimate MSSQL clients from accessing the server.	CICDDoS2019
SYN Attack	The SYN flood attack is one of the most exploited DDoS attacks associated with the vulnerability in the TCP-three-way handshake phase of the TCP protocol that is used by almost all network communications including the Internet. In this attack, the attacker sends a huge amount of SYN packets repeatedly until the target machine becomes unresponsive to legitimate users.	CICDDoS2019, BoT-IoT (named as TCP attack)
UDP Attack	Similar to TCP protocol, UDP protocol is used to deliver non-guaranteed delivery (i.e., packets can be lost) which is often used to deliver multimedia contents. Attackers send a massive number of UDP packets to random ports of a victim’s machine. This results in the network of the victim becoming exhausted or malfunctioning.	CICDDoS2019, BoT-IoT
HTTP Attack	Attackers overwhelm a victim’s server by opening and maintaining a massive number of simultaneous HTTP connections between the attacker and the victim.	CIC-IDS2017

**Table 4:** Training hyper-parameters (both autoencoder and MLP classifier).

Parameters	Values	Description
Batch Size	64	The number of training examples in one
		forward/backward pass.
Learning rate	0.001	Learning rate is used in the training of
		neural networks range [0.0, 1.0].
Optimizer	Adam [35]	a stochastic gradient descent method,
		an argument required for compiling model.
Epoch	20	Total iterations for the entire dataset.
Validation split	0.2	Validation ratio in training.

**Table 5:** Performance outcomes.

Dataset	Class	Precision	Recall	*F*1-*Score*
CICDDoS2019	BENIGN	0.91	0.99	0.95
	LDAP	0.91	0.99	0.95
	MSSQL	0.98	0.94	0.96
	NetBIOS	1.00	1.00	1.00
	Syn	1.00	1.00	1.00
	UDP	0.96	0.97	0.97
CIC-IDS2017	BENIGN	0.99	1.00	0.99
	DDoS	0.99	0.90	0.94
	DoS GoldenEye	0.97	0.92	0.94
	DoS Hulk	0.92	0.95	0.93
	DoS Slowloris	0.99	0.84	0.91
BoT-IoT	BENIGN	1.00	1.00	1.00
	DDoS	0.95	0.95	0.95
	DoS	0.94	0.94	0.94

**Table 6:** Comparison between our proposed system and other related work in smart transport system.

Metric	[3]	[4]	[6]	[7]	Our System
Blockchain based	Yes	Yes	Yes	Yes	Yes
Smart contract	Yes	No	Yes	Yes	Yes
Security	Yes	Yes	Yes	Yes	Yes
Trust	Yes	Yes	Yes	Yes	Yes
Integrity	Yes	Yes	Yes	Yes	Yes
DDoS attack Detection	No	No	No	No	Yes
Availability	No	No	No	No	Yes

## Data Availability

Not applicable.
